# Cervical cancer screening uptake and determinant factors among women in Ambo town, Western Oromia, Ethiopia: Community‐based cross‐sectional study

**DOI:** 10.1002/cam4.4369

**Published:** 2021-10-27

**Authors:** Shewaye F. Natae, Digafe T. Nigatu, Mulu K. Negawo, Wakeshe W. Mengesha

**Affiliations:** ^1^ Department of Public Health College of Medicine and Health Sciences Ambo University Ambo Ethiopia; ^2^ Department of Nursing College of Medicine and Health Sciences Ambo University Ambo Ethiopia

**Keywords:** developing countries, early detection of cervical cancer, Ethiopia, uterine cervical neoplasms

## Abstract

**Background:**

Cervical cancer is the second most common cancer and the leading cause of cancer‐related death in Ethiopian women. About 77.6% of women died of 6294 new cases reported in 2019. Early screening for cervical cancer has substantially reduced morbidity and mortality attributed to it. In Ethiopia, most of the women visit the health facilities at the late stage of the disease in which the offered intervention is not promising. Therefore, we aimed to assess the level of cervical cancer screening uptake and its determinant among women of Ambo town, Ethiopia.

**Methods:**

Community‐based cross‐sectional study was conducted among 422 women aged 20–65 years. An interviewer‐administered questionnaire was used to collect the data. Data were analyzed using SPSS version 25. Estimates were presented using an odds ratio (OR) with 95% CI. Statistical significance was declared at a *p* value of <0.05.

**Results:**

In the present study, 392 women were participated giving a response rate of 93%. Only 8.7% (34) of the study participants were received cervical cancer screening in their lifetime. Being in the age group of 30–39 years (AOR = 3.2, 95% CI: 1.22, 8.36), having cervical cancer‐related discussions with a healthcare provider (AOR = 3.5; 95% CI: 1.17, 10.7), and knowing the availability of cervical cancer screening service (AOR = 2.8; 95% CI: 1.03, 7.87) were significantly associated with uptake of cervical cancer screening.

**Conclusion:**

In this study, cervical cancer screening uptake is very low. Our study identifies clues for determinants of cervical cancer screening uptake. Thus, further studies using a better study design might be helpful to explore determinants of low utilization of CC screening services and suggest an appropriate intervention that increases CC screening uptake in the study area.

## Graphical abstract

CC screening Uptake, Ambo, Ethiopia.

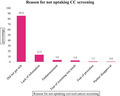

## 1 INTRODUCTION

Cervical cancer (CC) is cancer that arises from the cervix of the uterus. It is the fourth most prevalent cause of cancer deaths among women worldwide, accounting for 311,000 women's deaths annually.^1^, ^2^ More than 99% of cervical cancer cases result due to infection with high‐risk human papillomaviruses (HPV subtypes 16 and 18), which is transmitted through sexual contact.^3^, ^4^ Cervical cancer‐related mortality is reducing in affluent countries as the launch of the formal screening program.^5^ In contrast, the burden and mortality as a result of CC are increasing in low‐ and middle‐income countries (LMICs) due to the absence of well‐organized screening and HPV vaccination services.^4^, ^6^, ^7^ According to a 2015 report, of 270,000 cervical cancer deaths, 243,000 deaths were occurred in LMICs.^4^

Cervical cancer is the leading cause of cancer‐related mortality in African women. The prevalence of HPV infection in women with normal cytology was 24% in sub‐Saharan African (SSA) countries, followed by Eastern Europe (21%), and Latin America (16%).^8^ The disease is steadily increasing and result in more than 75,000 new cases and 50,000 deaths per year in SSA.^9^, ^10^ Of a total 443,000 women deaths predicted in 2030, about 90% of deaths will occur in SSA.^1^, ^11^ Ethiopia also shares a higher incidence of cervical cancer and mortality attributed to it. In 2010, it was estimated that 20.9 million women were at risk of developing cervical cancer with an estimated 4,648 and 3235 new cases and deaths, respectively.^12^ As to 2019 report of the country HPV information center, about 4,884 Ethiopian women died of 6294 new cases.^13^ Cervical cancer is the second most common cancer and the principal cause of cancer‐related death among Ethiopian women.^12^, ^14^

Unlike other reproductive organ cancers, cervical cancer is potentially preventable. Effective screening programs can lead to a significant reduction in the morbidity and mortality associated with CC.^5^, ^15^, ^16^ In high‐income countries, regular screening with cytology has been shown to lower the risk for developing invasive cervical cancer, through detecting precancerous changes.^6^ However, in LMICs, only about 5% of eligible women undergo cytology‐based screening in a 5‐year period.^6^, ^17^, ^18^ In almost all LMICs, cytology‐based services are limited to an advanced and higher level of health facilities or private laboratories in urban areas that are only accessed by the limited number of women.^6^, ^12^

In Ethiopia, due to lack of trained and skilled professionals, shortage of supplies, and well‐organized surveillance, scaling‐up and implementation of cytology‐based cervical cancer screening program is ineffective.^12^ However, visual inspection with acetic acid (VIA) is the commonest cervical cancer screening method that offered in most public health facilities of Ethiopia.^12^ It is an evidence‐based and affordable alternative approach for cervical cancer screening in low‐resource settings including Ethiopia.^6^, ^19^, ^20^ Studies have shown though it requires fewer resources and is feasible to carry out in low‐level health facilities, VIA is comparable sensitive to detect precancerous lesions to that of cervical cytology.^21^ A single round screening for CC by the VIA method reduced its incidence and mortality by 25% and 35%, respectively.^21^ In addition, VIA provides immediate results, consequently, promoting the linkage of screening with treatment.^19^ This “see and treat” method or single visit approach (SVA) ensures adherence to treatment soon after diagnosis and reduces the risk that women will get lost in the referral system.^19^

Although, WHO recommended CC screening at the age of 30–49 years^22^ and its screening at 21–65 years substantially reduces its incidence,^23^ advanced stage,^24^ and mortality^21^, ^23^, ^24^, ^25^; still, there are different barriers that hinder the utilization of the screening services. Socio‐economic status,^26^, ^27^, ^28^ public awareness,^28^, ^29^ fear of cancer and outcomes of screening,^28^, ^29^, ^30^ and healthcare access^27^, ^28^, ^30^ were barriers of cervical cancer screening uptake mainly in LMICs. Conversely, awareness of cervical cancer, family history of the disease, and experiencing the signs and symptoms of the disease were facilitators of its utilization.^28^

In the study area, most of the women visit health facilities at the later stage of the disease after it gets worsen than get screened at the earlier stage and prevents its consequence. Besides, the number of health facilities that offered the CC screening service is very limited. To date, there is no community‐based study yet conducted that assesses the level of CC screening utilization and its determinants in women of Ambo town. Thus, we aimed to investigate the level of screening service and its determinants in women of Ambo town, western Oromia, Ethiopia.

## 2 METHODS

### 2.1 Study area, period, and participants

A community‐based cross‐sectional study was conducted from December 01, 2017 to January 30, 2017, in Ambo town capital of west Shewa zone, western Ethiopia. The town is located 114 km to the west of Addis Ababa the capital of Ethiopia. According to the health planning, monitoring, and evaluation department report, the town has a total of 97,317 population, of which 18.6% of them were women of reproductive age group. In Ambo town, there are six subdistricts, one general and referral teaching hospital, two health centers, one Maternal and child health (MCH) clinic, and 20 private clinics that were providing different reproductive health services in the town. Currently, it is only the government hospital that provides cervical cancer screening services. All women aged between 20 and 65 years who are residing in Ambo town were the source population. Consequently, randomly selected women aged 20–65 years^23^ and who reside in the town during the data collection period were the sample population.

### 2.2 Sample size and sampling procedure

The sample size was calculated by using the single population proportion formula with the assumption of 95% CI, 50% proportion of women who uptake cervical cancer screening services, and 5% margin of error was used to obtain a sample size of 384, with adjustment of 10% nonresponse, the final sample size was 422.

$$n=\frac{1.96^{2}*\;0.5\left(1‐0.5\right)\;\;\;\;}{0.05^{2}}=384.$$

From six subdistricts of Ambo town, three subdistricts, namely subdistricts 01, 02, and 06, were selected by a simple random sampling technique. A total of 6705, 3513, and 3306 eligible women were found in subdistricts 01, 02, and 06, respectively. From those respective subdistricts, the sample size for this study was proportionally allocated; accordingly, 209, 109, and 103 women were selected from subdistricts 01, 02, and 06, respectively. Study participants were selected by systematic random sampling technique. The first household with the eligible subject was selected by the lottery method and then every 36th household with the eligible subject was included in the study. Women aged 20–65 years were interviewed from the selected households and if there were more than one woman in the household, the lottery method was used to select one.

### 2.3 Data collection tools and data collectors

The questionnaire was adapted from similar studies conducted in Gonder town,^31^ Jimma,^32^ and India.^33^ It was first prepared in English and then translated to the local language (Afan Oromo) and back‐translated into English by language experts to ensure its consistency. An interviewer‐administered pretested questionnaire was used for data collection. A pretest was done on 5% of women who live in different subdistricts to avoid contamination of information. The data were collected by six BSc Nurses and supervised by two MSc Nurses. Data collectors and supervisors were trained for 2 days before data collection. Personal identifiers were not included in the questionnaires to ensure the participants’ confidentiality. Supervisors and principal investigators checked the completeness of the questionnaires daily.

The tool covers variables such as sociodemographic characteristics, reproductive and sexual history, cervical cancer and screening knowledge, and cervical cancer screening uptake.

### 2.4 Operational definitions

#### 2.4.1 Cervical cancer screening uptake

The proportion of women who have ever been screened for cervical cancer at least once in their lifetime.^34^

### 2.5 Data processing and analysis

Data were coded, entered, and cleaned using Epi info version 3.5.1 and exported to SPSS version 25 statistical package for analyses. Descriptive statistics were computed and presented using tables and figures. The outcome variable is dichotomous coded as “1” when the respondents were screened for CC, otherwise “0.” Frequencies and proportions were calculated for a description related to socio‐demographic and other variables. Relationships between dependent and independent variables were investigated using the Binary logistic regression model. Those variables with *p*‐value below 0.25 in bivariate logistic regression were included in a multivariate logistic regression model for controlling potential confounding effects. The independent predictors for cervical cancer screening uptake are presented using an adjusted odds ratio (AOR) with its 95% CI. A statistical significance was established as *p* < 0.05.

## 3 RESULTS

### 3.1 Sociodemographic characteristics

A total of 392 women aged 20–65 years old were interviewed making a 92.9% response rate. The mean age of the study participants was 30.1 years (±9.1 SD). The majority (96.4%) of the respondents were from the Oromo ethnic group. About two‐thirds of the women were married, and 117 (29.8%) of them completed college and above. Nearly, half (48%) of the women were housewives in their occupation (Table 1).

**TABLE 1:** Sociodemographic characteristics of study participants in Ambo town western Oromia, Ethiopia, December–January 2017

Variable (*n* = 392)	Category	Frequency	Percentage
Age (years)	20–29	228	58.2
	30–39	109	27.8
	40–49	34	8.7
	>49	21	5.4
Ethnicity	Oromo	378	96.4
	Others^a^	14	3.6
Religion	Orthodox	151	38.5
	Protestant	228	58.2
	Others^b^	13	3.3
Marital status	Single	97	24.7
	Married	254	64.8
	Divorced	17	4.3
	Widowed	24	6.2
Educational status	Illiterate	67	17.1
	Attend primary	103	26.3
	Attend secondary	105	26.8
	College and above	117	29.8
Occupation	House wife	88	48
	Private employee	42	10.7
	Governmental employee	80	20.4
	Daily laborer	38	9.7
	Student	44	11.2
Income	No income	37	9.4
	100–1000 ET birr	157	40.1
	>1000 ET birr	198	50.5

^a^ Amhara and Gurage.

^b^ Catholic, and waqefata, Musilim.

### 3.2 Reproductive characteristics of the respondents

Around 256 (65.3%) of the participants had a single sexual partner, whereas the rest (34.7%) had multiple sexual partners in their lifetime. Two hundred and forty‐three (62.0%) of the participants had two and above pregnancies (Table 2).

**TABLE 2:** Reproductive characteristics of the study participants in Ambo town, western Oromia, Ethiopia, December–January 2017

Variable (*n* = 392)	Category	Frequency	Percent
Age at first intercourse	<18 years	159	40.6
	≥18 years	233	59.4
Have you been pregnant	Yes	324	82.7
	No	68	17.3
Number of pregnancy	<2	149	38.0
	≥2	243	62.0
Have you give birth	Yes	300	76.5
	No	92	23.5
Number of children	<2	156	39.8
	≥2	236	60.2
Lifetime number of sexual partner	Single partner	256	65.3
	Multiple partner	136	34.7
History of abortion	Yes	47	12.0
	No	345	88.0
History of contraceptive use	Yes	208	53.1
	No	184	46.9

### 3.3 Knowledge of cervical cancer

**FIGURE 1:**
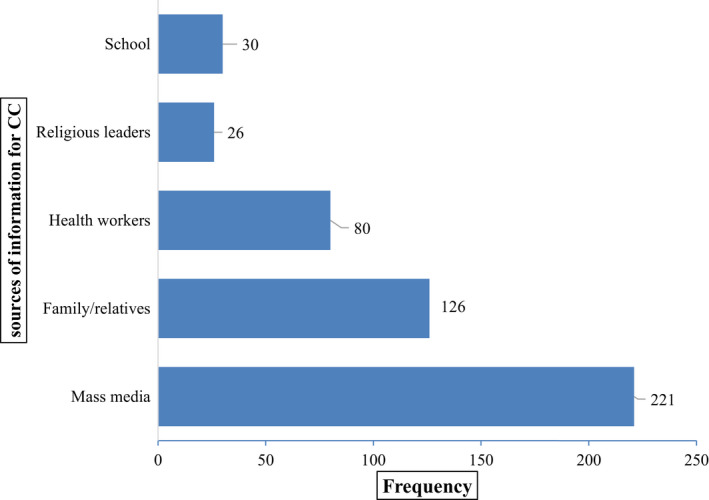
Sources of information of cervical cancer reported by the study subjects (multiple responses were considered; thus the sum might be more than sample size)

Three hundred and eight (78.6%) subjects have ever heard of cervical cancer; mass media was the most source of information reported by study subjects (Figure 1). Of the study participants, only 180 (45.9%) of them know of cervical cancer, whereas more than half (54.1%) of the women did not know cancer of the cervix. Most (68.3%) of the women reported unusual vaginal bleeding as the symptom of CC, whereas the rest reported vaginal discharge mixed with blood (42.2%), dyspareunia (32.2%), pelvic pain (12.8%), and the least reported back pain (2.2%) as the symptom of CC. Regarding the preventive mechanisms of cervical cancer, only 45 (11.5%) and 15 (3.8%) of the women knew HPV vaccination and screening for cervical cancer prevent CC, respectively.

### 3.4 Cervical cancer screening uptake and reason for not getting screened

**FIGURE 2:**
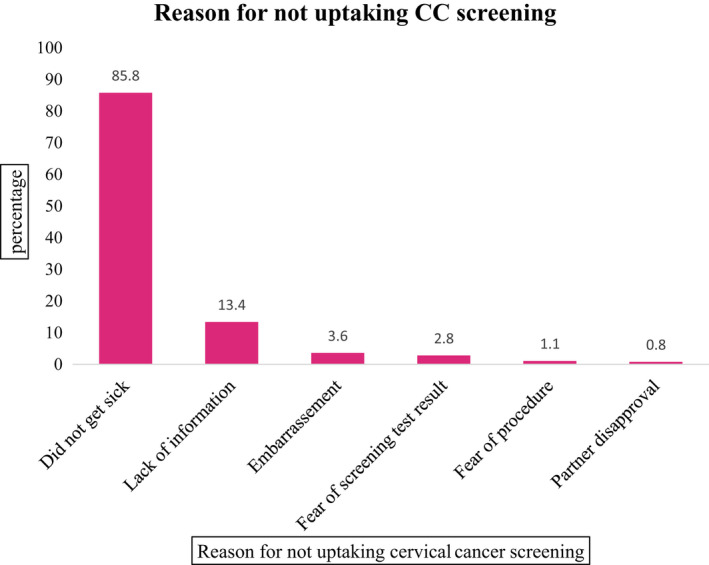
Main reason for not uptaking cervical cancer screening by women of Ambo town, western Oromia, Ethiopia, December–January 2017

Only 34 (8.7%) of the women had ever screened for cervical cancer in their lifetime. However, 247(63.0%) of the women had knowledge about cervical cancer screening. Visual inspection with acetic acid (VIA) (44.1%) was the most screening type reported by respondents who had ever screened for cervical cancer, followed by a blood test (35.3%), and papanicolaou (Pap) test (20.6%). Not getting sick of CC is the main reason for the nonutilization of CC screening services (Figure 2).

### 3.5 Factors associated with uptake of cervical cancer screening

In bivariate logistic regression analysis, age, knowing the availability of the screening service in the public hospital, history of early sexual initiation, know the consequence of advanced cervical cancer (metastasis and bleeding), knowledge of CC screening, and discussion on cervical cancer with healthcare providers were statistically significant with the uptake of cervical cancer screening. However, in multivariable logistic regression analysis, age, knowledge on the consequence of advanced cervical cancer (metastasis and bleeding), knowledge of CC screening, and discussion with healthcare providers on CC screening were remained significantly associated with cervical cancer screening uptake (Table 3).

**TABLE 3:** Determinants of cervical cancer screening uptake among women of Ambo town, western Oromia, Ethiopia, December–January 2017

Variables	Total	Cervical cancer screening uptake		COR, 95%CI	AOR, 95% CI
		Yes	No		
Age (years)					
20–29	228	10 (4.4)	218 (95.6)	1.00	1.00
30–39	109	14 (12.8)	95 (87.2)	3.2 (1.4,7.5)*	3.2 (1.2,8.4)*
40–49	34	6 (17.6)	28 (82.4)	4.7 (1.6,13.8)*	4.8 (1.4,16.4)*
>49	21	4 (19.0)	17 (81.0)	5.1 (1.5,18.1)*	4.3 (0.9,19.6)
Give birth to many children					
Yes	80	15 (18.8)	65 (81.3)	3.6 (1.7,7.4)*	2.7 (1.3,5.9)*
No	312	19 (6.1)	293 (93.9)	1.00	1.00
Screening service availability					
Yes	48	8 (16.7)	40 (83.3)	2.5 (1.0,5.8)*	1.9 (0.8,4.8)
No	344	26 (7.6)	318 (92.4)	1.00	1.00
Knew metastasis					
Yes	53	11 (20.8)	42 (79.2)	3.6 (1.6,7.9)*	2.9 (1.2,6.9)*
No	339	23 (6.8)	316 (93.2)	1.00	1.00
Knew bleeding					
Yes	30	9 (30.0)	21 (70.0)	5.8 (2.4,13.9)*	3.1 (1.2,8.3)*
No	362	25 (6.9)	337 (93.1)	1.00	1.00
Knowledge on CC screening					
Yes	247	29 (11.7)	218 (88.3)	3.7 (1.4,9.9)*	2.8 (1.0,7.9)*
No	145	5 (3.4)	140 (96.6)	1.00	1.00
Knowledge on CC					
Yes	180	19 (10.6)	161 (89.4)	1.6 (0.8,3.2)	1.0 (0.4,2.2)
No	212	15 (7.1)	197 (92.9)	1.00	1.00
Discussed CC with HCP					
Yes	31	6 (19.4)	25 (80.6)	2.9 (1.1,7.5)*	3.5 (1.2,10.7)*
No	361	28 (7.8)	333 (92.2)	1.00	1.00

Abbreviations: CC, cervical cancer; HCP, healthcare provider.

* *p*‐value <0.05.

This study indicated that those women who were in the age group of 30–39 years and 40–49 years were 3.2 times (AOR = 3.2; 95% CI [1.22,8.36]) and 4.8 times (AOR = 4.8;95% CI [1.42,16.41]) more likely to uptake cervical cancer screening compared with those women whose ages between 20 and 29 years, respectively. Those women who knew giving birth to many children as a risk factor for cervical cancer were 2.7 times (AOR = 2.7; 95% CI [1.26, 5.98]) more likely to utilize cervical cancer screening compared with their counterparts. Women who had knowledge about cervical cancer screening were 2.8 times (AOR = 2.8; 95% CI [1.03, 7.87]) more likely to uptake cervical cancer screening than their counterparts. Furthermore, the present study found that knowledge on the consequences of advanced cervical cancer increases the uptake of cervical cancer screening by the study subjects. Those women who knew metastasis and bleeding were the consequence of advanced cervical cancer were 2.9 times (AOR = 2.9; 95% CI [1.20, 6.95]) and 3.1times (AOR = 3.1; 95% CI [1.16, 8.29]) more likely to utilize the CC screening service than their counterparts, respectively.

## 4 DISCUSSION

A total of 392 women have participated in the present study gives a response rate of 92.9%. In this study, despite a relatively high level of respondents, having knowledge of cervical cancer (45.9%) and its screening (63.0%), only 8.7% of the women utilized cervical cancer screening services in their lifetime. The finding of the present study is lower than the former studies conducted in Hosanna town (10%),^35^ Dessie town (11%),^36^ Mekele town (20%),^37^ and Debremarkos town (21%) of Ethiopia.^38^ The low level of cervical cancer screening uptake in our study despite a relatively high level of knowledge of cervical cancer and its screening service might be attributed to an inadequate number of health facilities that provide cervical cancer screening service, health‐seeking behavior of the study participants, and knowledge of the women of the preventive measures of cervical cancer.

In the study area at the time of data collection, only one hospital provide the cervical cancer screening service. Though the women were knowledgeable about cervical cancer and its screening service, the service is not easily accessible by all eligible women due to an inadequate number of service provision centers in the study area. Besides, the health‐seeking behavior of women, particularly of preventive services is very low. In the present study, only 3.8% of the women knew as cervical cancer screening prevents cervical cancer, this indicates as there is a huge gap between knowledge of CC and its prevention methods. Likewise, our study also reveals that not getting sick is the main reason for the nonutilizing of cervical cancer screening. This highlighted if they perceive that they are healthy and did not seek the services as early as possible rather they seek it after they experienced the clinical feature of the diseases due to its advancement. Our study is in line with the studies conducted in Uganda^39^ and Ethiopia^40^, ^41^ that point out most women present with cervical cancer at an advanced stage due to poor health‐seeking behavior.

Another study^42^ also indicated that 17% of undergraduate female University students underwent cervical cancer screening in their lifetime. The level of screening from this study was also higher than that of the present study; the reason might be due to the variation of awareness level among the University students and women in the general community. Studies showed that women who had a higher level of education^41^, ^43^, ^44^ and knowledge on cervical cancer,^35^, ^36^, ^37^ and its screening^37^, ^44^ were more utilized the screening services than their counterparts. Conversely, the cervical cancer screening level of the present study was higher than the community‐based studies conducted among women in Finote‐Selam city (7.3%), northwest Ethiopia,^45^ in Butajira district, (2.3%), southern Ethiopia,^41^ and in Debre Markos town (5.4%), northwest Ethiopia.^44^ The inconsistencies might be due to the difference in study settings and age groups included in the studies.

The current study revealed that age (being within the age group of 30–39 years and 40–49 years), knew metastasis and bleeding are the consequences of advanced CC, and knowledge on CC screening, discussion with healthcare providers on CC screening, and give birth to many children were predictors of cervical cancer screening uptake. Women in the age group of 30–39 years (3.2 times) and 40–49 years (4.8 times) are more likely to be screened for cervical cancer compared with those aged between 20 and 29 years. The finding was in line with studies conducted in public hospitals found in the Tigray region^46^ and Mekele town^37^ that reported being aged between 30 and 39 years were two times more likely to utilize screening services than those who were 21–29 years old. Moreover, the likelihood of cervical cancer screening uptake is increasing as age increased. Those women whose age ranges 40–49 years were 4 times,^46^ 3.1 times,^44^ and 2.4 times^47^ more likely to utilize the screening services compared with 21–29 years old. Also studies conducted in Dessie town,^36^ Debre Markos town,^38^ and Finote Selam city^45^ revealed that women aged between 34 and 49 years were 6.0 times, 3.2 times, and 2.8 times more likely to be screened for CC than the younger age group, respectively.

Furthermore, discussing with healthcare providers about cervical cancer screening is also increasing its utilization; those women who had ever discussed with the healthcare providers about CC screening were 3.5 times more likely to be screened than their counterparts. This finding is also supported by studies conducted in Debre‐Markos town and Bahir‐Dar city,^38^, ^48^ which revealed that being informed about CC screening by health professionals increases its utilization by 6.8 folds.

Former studies also indicated that knowledge on cervical cancer screening was increased CC screening uptake by 2.36 fold^37^ and 4.02 fold.^44^ The present study also in accordance with those studies; women who had knowledgeable of cervical cancer screening were 2.8 times more likely to uptake cervical screening than those who did not know the screening. Our study also reveals knowledge of metastasis and bleeding due to advanced CC increases cervical cancer screening utilization. Those women who knew metastasis (2.2 times) and bleeding (3.1 times) are occurred due to advanced CC were more likely to utilize CC screening than those who did not know the advanced cervical cancer consequence.

Our study identifies the main reason for not up‐taking cervical cancer screening. Self‐perceived health (85.8%), lack of information (13.4%), and fear of screening test results (2.8%) were the frequently reported reason for the nonutilization of CC screening by the study participants. This result is also in harmony with studies conducted in Hossana, southern Ethiopia,^49^ and Addis Ababa^50^ that indicated a lack of information and awareness were barriers to CC screening uptake. A cluster‐randomized trial study conducted in Butajira district also revealed self‐assertion of being healthy and fear of screening were the main reason for nonutilizing screening services.^51^

Altogether, the knowledge level of study participants regarding the preventive measures of cervical cancer is low; only 3.8% and 11.5% of women reported screening for cervical cancer and HPV vaccine prevents cervical cancer, respectively. A Mukama T et al. found a higher level of CC preventive measures, early screening (46%), and vaccination for HPV (33.3%)^52^ than the present study. The inconsistencies might be attributed to sample size and study setting. In Ethiopia, the HPV vaccine was launched for the first time in 2018 with the support of the Global Alliance for vaccine and immunization (GAVI) for girls who are 14 years old.^53^ Currently, the vaccine was delivered through a school‐based approach to access all eligible girls of Ethiopia.^53^ Due to the vaccination against HPV being launched 1 year later after the current study, the women who participated in the present study were not get vaccinated.

Though Ethiopian women are willing to vaccinate their children for HPV^54^, ^55^ still there are challenges that affect its effective utilization. Ineffectiveness of the recently provided vaccine (HPV2 and HPV4) for other prevalent strains of HPV (HPV‐52,^56^, ^57^ HPV‐56,^57^, ^58^ HPV‐58,^57^ and HPV‐31^57^) in Ethiopia, high vaccine costs, inadequate delivery system, and lack of community involvement to create awareness about CC and early screening tools are challenges that hinder effective utilization HPV vaccination in Ethiopia.^31^, ^40^, ^59^, ^60^, ^61^ Therefore, targeting the common barriers of HPV vaccination for its effective utilization and increasing the accessibility of CC screening through mainstreaming the cervical cancer screening service in all units of the health facility to reach more eligible women and increase their awareness level, decentralization of cervical cancer screening services at all levels of health facilities, and offering the CC screening for free at all health facilities are of paramount importance for reducing morbidity and mortality attributed to cervical cancer in Ethiopian women.

### 4.1 Limitation of the study

The descriptive nature of the study design might not be strong as other analytic or experimental study designs to reach on sound conclusion and valid recommendations that intervene to increase CC screening uptake.

## 5 CONCLUSION

In this study, cervical cancer screening uptake is very low. Our study identifies clues for determinants of cervical cancer screening uptake. Thus, further studies using a better study design might be helpful to explore determinants of low utilization of CC screening services and suggest an appropriate intervention that increases CC screening uptake in the study area.

## CONFLICT OF INTERESTS

The authors declare that they have no competing interests.

## AUTHOR CONTRIBUTIONS

Shewaye Fituma Natae: conceptualization, analysis, and write up and manuscript preparation. Digafe Tsegaye Nigatu: analysis, interpretation of the data, and review the manuscript. Wakeshe Willi Mengesha: involves in the preparations of the manuscript and reviewing the paper. Mulu kitaba Negawo: involves in the preparations of the manuscript and reviewing the paper. All authors read and approved the final manuscript.

## ETHICS APPROVAL AND CONSENT TO PARTICIPATE

The study was approved by the ethical Review committee of the College of Medicine and Health Sciences (CMHS‐ERC) of Ambo University, Ethiopia and all performed procedures were in accordance with the 1964 Helsinki declaration and its later amendments. Written informed consent was sought from all participants after the aim of the study was introduced. Confidentiality of the gathered information was assured to the interviewee.

## Data Availability

The data sets used and/or analyzed during the current study are available from the corresponding author on reasonable request.
